# Characterization of a Stable, Metronidazole-Resistant *Clostridium difficile* Clinical Isolate

**DOI:** 10.1371/journal.pone.0053757

**Published:** 2013-01-17

**Authors:** Tarah Lynch, Patrick Chong, Jason Zhang, Romeo Hizon, Tim Du, Morag R. Graham, Daniel R. Beniac, Timothy F. Booth, Pamela Kibsey, Mark Miller, Denise Gravel, Michael R. Mulvey, Canadian Nosocomial Infection Surveillance Program (CNISP)

**Affiliations:** 1 National Microbiology Laboratory, Public Health Agency of Canada, Winnipeg, Manitoba, Canada; 2 Victoria General Hospital, Victoria, British Colombia, Canada; 3 Jewish General Hospital, Montreal, Quebec, Canada; 4 Public Health Agency of Canada, Ottawa, Ontario, Canada; Institut National de la Recherche Agronomique, France

## Abstract

**Background:**

*Clostridium difficile* are Gram-positive, spore forming anaerobic bacteria that are the leading cause of healthcare-associated diarrhea, usually associated with antibiotic usage. Metronidazole is currently the first-line treatment for mild to moderate *C. difficile* diarrhea however recurrence occurs at rates of 15–35%. There are few reports of *C. difficile* metronidazole resistance in the literature, and when observed, the phenotype has been transient and lost after storage or exposure of the bacteria to freeze/thaw cycles. Owing to the unstable nature of the resistance phenotype in the laboratory, clinical significance and understanding of the resistance mechanisms is lacking.

**Methodology/Principal Findings:**

Genotypic and phenotypic characterization was performed on a metronidazole resistant clinical isolate of *C. difficile*. Whole-genome sequencing was used to identify potential genetic contributions to the phenotypic variation observed with molecular and bacteriological techniques. Phenotypic observations of the metronidazole resistant strain revealed aberrant growth in broth and elongated cell morphology relative to a metronidazole-susceptible, wild type NAP1 strain. Comparative genomic analysis revealed single nucleotide polymorphism (SNP) level variation within genes affecting core metabolic pathways such as electron transport, iron utilization and energy production.

**Conclusions/Significance:**

This is the first characterization of stable, metronidazole resistance in a *C. difficile* isolate. The study provides an in-depth genomic and phenotypic analysis of this strain and provides a foundation for future studies to elucidate mechanisms conferring metronidazole resistance in *C. difficile* that have not been previously described.

## Introduction

*Clostridium difficile* infection (CDI) is the most common cause of healthcare-associated infectious diarrhea. Disease severity can range from self-limiting diarrhea to pseudomembranous colitis which can be associated with additional complications leading to increased mortality rates [1], 2. Prevalence of CDI has increased dramatically with emergence of the hypervirulent NAP1/027(North American pulse-field type 1, PCR ribotype 027) strain in 2003 [3]–[6]. Laboratory diagnosis involves detection of toxin A and B in stool samples by enzyme immunoassay and/or molecular detection [7]. Stool culture remains the most sensitive diagnostic however owing to the slow turnaround time it is more often used for epidemiological studies rather than patient diagnosis [7]. Antibiotic susceptibility testing on *C. difficile* is not routinely performed since the assay is too time consuming. The Clinical Laboratory Standards Institute (CLSI) susceptibility breakpoints are based on therapeutic levels in the serum and not the intestinal lumen (site of action) and there is poor correlation between *in vitro* susceptibility and *in vivo* efficacy [8].

Metronidazole is the recommended treatment for mild to moderate CDI, while vancomycin is reserved for more severe cases owing to cost and concerns of vancomycin-resistant nosocomial infections [9], [10]. Fidaxomicin is a new class of narrow spectrum macrocyclic antibiotics recently licensed for treatment of CDI but is not yet widely used [11]. The pressure to develop alternative therapies for CDI stems from the incidence of disease recurrence that can follow treatment with metronidazole, vancomycin or fidaxomicin (15–35%) [9], [11]–[13] and the fear that development of resistance to one of more of these agents will limit our ability to treat CDI.

Recent studies support the general assumption that most *C. difficile* isolates remain susceptible to vancomycin and metronidazole [14]–[17] however metronidazole and vancomycin resistance has been reported [18]–[21]. A study from Spain in 2002 reported 3.1% of *C. difficile* isolates with intermediate resistance to vancomycin and 6.3% to metronidazole (MIC value≥16 mcg/ml breakpoint) [22]. Such reports of metronidazole resistance in *C. difficile* have all observed loss of the resistant phenotype after passaging or low temperature storage [19], [23], [24]; moreover, there was only one study that found resistance in epidemic, NAP1/027-typed isolates [20]. The transient nature of the resistant phenotype has made further investigation of metronidazole resistance mechanisms difficult to pursue in *C. difficile*.

Metronidazole resistance mechanisms have been extensively investigated in other pathogenic bacteria such as *Bacteroides fragilis* and *Helicobacter pylori* [25]–[28]. Multiple metronidazole resistance mechanisms have been described, even within a single bacterial species. For example, mechanisms described for *B. fragilis* include the presence of *nim* genes, which encode 5-nitroimidazole reductases that convert metronidazole to a non-toxic amino derivative [29]–[31], overexpression of the DNA repair protein, RecA [28] and disruption of the electron transport chain [32]. Similarly, mechanisms in *H. pylori* that potentially contribute to metronidazole resistance have been described involving mutations in the *rdxA* and *frxA* nitroreductase genes [33]–[35] and also overexpression of *recA* [36]. Additionally, overexpression of the *hefA* bacterial efflux pump [37] and mutations within the ferric uptake regulator (*fur*) gene, which is responsible for responding to reactive oxygen species and iron uptake [25], [38] have also been reported to confer metronidazole resistance in *H. pylori*.

In 2009, our laboratory isolated a *C. difficile*, NAP1 strain displaying metronidazole resistance (MIC>32 mcg/ml). After passaging the isolate on media containing sub-inhibitory concentrations of metronidazole (8 mcg/ml), we successfully cultured a stably maintained isolate with the resistant phenotype notably present after freeze-thawing (in contrast to prior reports in which the metronidazole resistance phenotype was lost after freezing or cold storage). To investigate further, we sequenced the genome of the stable-metronidazole resistant isolate as well as a second sub-population from the same clinical sample that had reverted after freezer storage to become only partially susceptible to metronidazole. The aim of the current work is to present an initial genomic and phenotypic characterization of the metronidazole-resistant and reduced susceptible isolates in the pursuit of elucidating the resistance mechanisms and potential clinical implications associated with *C. difficile* metronidazole resistance.

## Materials and Methods

### Strain Isolation

Strain CD26A54 was isolated from a stool sample collected under the Canadian Nosocomial Surveillance Program (CNISP) which continuously monitors health-care acquired infections across Canada. Data collection was observational and considered a routine component of institutional infection prevention and control practices under provincial legislation, therefore informed consent was not required [39]. CNISP collected data indicated the patient received standard metronidazole treatment however multiple courses were administered due to recurrent CDI.

The stool sample was processed as previously described [40] however, beginning in 2009, suspensions were also planted on *C. difficile*-moxalactam-norfloxacin (CDMN) agar (OXOID, Nepean, ON, Canada) supplemented with 8 mcg/ml metronidazole as an additional screen for potential metronidazole resistance. Growth was observed on the CDMN+metronidazole plate after anaerobic incubation for 48 hours at 35–37°C. A colony was passaged onto Brucella agar supplemented with 10 mcg/ml vitamin K, 5 mcg/ml haemin, 5% laked sheep blood (BAKHS; BD, Mississauga, ON, Canada)+8 mcg/ml metronidazole. A sub-population from this plate was stored at −80°C while the other sub-population was further passaged on BAKHS+8 mcg/ml metronidazole (approximately 12 passages). The subpopulation that was immediately frozen subsequently tested susceptible to metronidazole (although slightly elevated MIC compared to wild type *C. difficile* isolates regularly tested in our laboratory) and was designated as CD26A54_S (**S**usceptible), while the subpopulation that was continually passaged on metronidazole-containing agar retained the resistant phenotype even after the freeze-thaw process, it is referred to herein as CD26A54_R (**R**esistant).

The NAP1 *C. difficile* strain, VLOO13 was used as a control strain in the present experiments. Our laboratory confirmed that VLOO13 had an indistinguishable NAP1 PFGE pattern and toxin genotype to the CD26A54_R and CD26A54_S isolates, while being geographically unrelated, and has also never demonstrated reduced susceptibility to metronidazole. VLOO13 was provided in kind by Dr. T. Louie (University of Calgary, Calgary, Canada).

### Molecular characterization of *C. difficile*

DNA for PCR analysis was prepared using InstaGene Matrix (Bio-Rad, Richmond, CA, USA). Multipex PCR was employed to detect the Toxin A (*tcdA*), Toxin B (*tcdB*), Binary toxin (*cdtB*), the negative regulator of toxin production (*tcdC*) and the triose phosphate isomerase (*tpi*) genes [41], [42].

Pulsed-field gel electrophoresis (PFGE) typing was performed with *Sma*I-digested DNA following a validated protocol found elsewhere [43]. BioNumerics software version 4.0 (Applied Maths, Austin, TX, USA) was used to analyze and compare the gel images. Cluster analysis parameters included the Dice coefficient and the unweighted-pair group method with arithmetic means [20]. Isolates that produced PFGE patterns with at least 80% homology were considered to be within the same PFGE type [20].

### Culturing methods and quantitation

All experiments were performed on strains that had been passaged three times on BAKHS agar after being removed from −80°C storage to maintain consistency between technical replicates. Growth curves were performed in Brain Heart Infusion (BHI) broth (BD, Mississauga, ON, Canada) at 37°C in an anaerobic chamber (Coy Laboratory Products Inc, Grass Lake, MI). The turbidity for all overnight starter cultures was measured with a spectrophotometer (Eppendorf, Mississauga, ON, Canada) at OD 600 nm and normalized before inoculating fresh growth curve tubes to minimize variation at time point 0. Quantitation of vegetative cells relative to spores was performed following a protocol described elsewhere [44] using alcohol shock to select for spores and subsequent serial dilutions and enumeration on CDMN agar.

### *C. difficile* preparation for scanning electron microscopy

All samples were filtered using 13 mm diameter SPI–pore polycarbonate track etch filters with 100 nm pores (SPI supplies, West Chester, Pennsylvania, USA), held in 13 mm Swinnex® filter holders (Millipore, Billerica, Massachusetts, USA). All syringe filtering was performed using a Legato 200 syringe pump (KD Scientific, Holliston, Massachusetts, USA) operated with a flow rate of 1 ml/minute to ensure reproducible filtration conditions. When starting with fresh bacterial samples grown an agar plates, two loopfuls of bacteria were mixed in 800 µl of fixative (1% paraformaldehyde, 2% glutaraldehyde) generating a turbid suspension. The subsequent filter preparation of all the specimens was done in a fume hood, using Lure-Lok® syringes to pass all fluids through the filter assembly. Sample processing was conducted as follows: using a 2 ml syringe, 2 ml of PBS was passed through the apparatus to wet the filter. Next, 0.2 ml of the bacterial suspension was applied to the filter using a 1 ml syringe, followed by a 5 ml wash with PBS done using a 5 ml syringe. Then using 2 ml syringes, the filter was washed with 2 ml 50% ethanol, 2 ml 70% ethanol, 2 ml 85% ethanol, 2 ml 95% ethanol, and 2 ml 100% ethanol. The filter apparatus was then disassembled and the filter was allowed to air dry.

Once dry, the filter was cut and mounted onto an SEM stub. First, a double-sided adhesive carbon disc was stuck to the metal stub, and then the filter was mounted to the carbon disc, bacteria side up. Silver flash paint was then used to create a conductive contact between the stub and the filter paper. 1 cm of 0.2 mm diameter gold wire was measured, cut and wound on the filament of an Agar 208 Turbo Carbon Coater evaporator unit (Agar Scientific, Stansted, England). The vacuum was turned on, and once a vacuum was generated the low tension was gradually increased to evaporate the gold onto the sample.

Specimens were imaged in a Scanning Electron Microscope (JEOL CarryScope, JEOL Ltd., Tokyo, Japan) operated at 6 kV, with a spot size of 20, a 9 mm working distance, and at nominal instrument magnification of ×3,000. Digital images were acquired using the secondary electron detector.

*C. difficile* length measurements (*n*≈1000/dataset) were made using the Image J software package [45]. Using the segmented line tool, and the analyze/measure function for all of the SEM images of *C. difficile*. The measurements that were made in image J were then collated, analyzed, and plotted, using Microsoft Excel.

### Antimicrobial susceptibility testing

Susceptibility to metronidazole, clindamycin, vancomycin, rifampicin, moxifloxacin and tigecycline were performed using Etest® strips (bioMérieux, Solina, Sweden) with standard methods described elsewhere [46]. Briefly, the standard protocol involved BAKHS agar plates, incubated for 48 hours at 37°C under anaerobic conditions. Additional susceptibility testing to metronidazole by agar dilution was performed on the original isolate from stool to confirm the Etest® results. Results are reported as minimum inhibitory concentrations (MIC) in mcg/ml of the antibiotic being tested. Best efforts were made to ensure reproducibility of the Etest® assays by consistently testing strains at the same passage number, using BAKHS agar plates within 2 weeks of production and maintaining a proper anaerobic environment with an O_2_/H_2_ gas analyzer (COY Laboratory Products, Grass Lake, Michigan, USA) installed in the anaerobic chamber. As per CLSI guidelines, control ATCC strains were always used in parallel with test samples to validate our results [46].

To test for heteroresistance towards metronidazole, Etest® assays were again set up according to the manufacturer's instructions, however they were incubated for an extended period of time to observe the presence or absence of slower growing satellite colonies within the clear ellipse. A similar protocol has been described previously [23], however the incubation time was modified as we empirically established that 96 hours incubation was sufficient to determine heteroresistance; longer time points (up to 10 days) did not reveal new colonies in our laboratory.

### Whole-genome sequencing (WGS)

Draft genomes were acquired for the CD26A54_R and CD26A54_S isolates via combined use of single-end shotgun pyrosequencing reads (GS FLX-Titanium; Roche Diagnostics, Indianapolis, IN, USA) and paired-end, 100 bp Illumina reads (GAIIe; Illumina, San Diego, CA, USA; Sequenced at Eurofins MWG Operon, Huntsville, AL, USA). Pyrosequencing yielded an average 22X and 24X coverage while the Illumina data increased the coverage to 228X and 323X average coverage of the CD26A54_S and CD26A54_R genomes, respectively. The combined sequence data provided an estimated >99% coverage of each genome. Publically available NAP1 genomes were employed for comparative genomic analyses with the CD26A54_R and CD26A54_S strains [47], [48] (Table 1).

**Table 1:** Summary of genomes used in comparative analyses.

Strain	Year	Location	Source	Assembly	Genome size (nt)	No. of contigs	Reference	NCBI accession
CD26A54_R	2009	B.C., Canada	Human	draft	4,166,900	4	this study	PRJNA172829
CD26A54_S	2009	B.C., Canada	Human	draft	4,166,979	6	this study	PRJNA172828
QCD-66c26	2007	QC, Canada	Human	draft	4,094,363	32	44	NZ_CM000441
R20291	2006	UK	Human	complete	4,191,339	1	45	NC_013316
QCD-37×79	2005	ON, Canada	Human	draft	4,092,698	45	44	NZ_CM000658
QCD-97b34	2004	Nfld, Canada	Human	draft	3,998,408	60	44	NZ_CM000657
QCD-32g58	2004	QC, Canada	Human	draft	4,109,689	16	44	NZ_CM000287
BI-1	1988	USA	Human	draft	4,118,573	66	44	NC_017179
CD196	1985	France	Human	complete	4,110,554	1	45	NC_013315

### Bioinformatic analyses

GS-FLX reads were assembled *de novo* using Newbler version 2.5.3 (Roche Diagnostics). The Illumina GAIIe reads were then mapped to the Newbler assembled GS FLX contigs to correct potential homopolymer errors using CLC Genomics Workbench version 4.9. The resultant contigs were then ordered using the NAP1 reference sequence R20291 (GenBank: NC_013316.1 [48]) and some gap closure was completed by integration of PCR-generated products using Staden Gap4 software [49]. These ordered contigs were connected with a linker sequence containing stop codons in all 6 translation reading frames creating high quality pseudogenomes for annotation and further downstream analyses. Automated annotation was accomplished using an in-house, modified version of GenDB version 2.2 [50].

CD26A54_R and CD26A54_S were compared with other publically available NAP1 strains by generating a BLAST atlas (listed in Table 1). BLASTn was run with an expected cutoff value of 1×10^−10^ and a percent identity cutoff value of 80% for all nucleotide segments greater than 100 bp and the results visualized in GView [51]. Analysis for single nucleotide polymorphisms (SNP) and insertion/deletion (indel) mutations were performed using BWA version 6.1 [52] to map the Illumina GAIIe reads to the R20291 reference sequence followed by data parsing of the alignments (SAMtools version 1.18; [53]) and variant calling with VCFtools version 1.8 [54].

### Statistical Analysis

Growth curves were analyzed with a two-way ANOVA and Tukey posttest. The Kruskal-Wallis test for variance with Dunn's posttest was used to analyze the SEM length measurement and metronidazole MIC data as normality could not be assumed for these distributions. All analyses were conducted in PRISM 5 statistical software (GraphPad Software, La Jolla, CA, USA).

## Results

### Molecular identification of the study strains

All three strains in the study, CD26A54_R, CD26A54_S and VLOO13 were typed as NAP1, pattern 001 by pulse-field gel electrophoresis (PFGE). The PCR toxin analyses were typical of NAP1 strains, being positive for all toxins (*tcdA*, *tcdB*, *cdtB*), the *tpi* and *tcdC* genes, with the characteristic 18 bp deletion in the *tcdC* gene [55]. CD26A54_R, CD26A54_S and VLOO13 were indistinguishable by these standard typing methods. Both CD26A54 subpopulations were considered clonal, as they originated from a colony isolated from a single stool sample despite having subsequent divergent environmental exposures. Specifically, the CD26A54_S subpopulation was immediately frozen after isolation causing this strain to subsequently become susceptible to metronidazole under standard laboratory conditions. In contrast, CD26A54_R was continually passaged in the presence of selective antibiotic pressure, likely causing adaptive changes that made it stably resistant to metronidazole.

### Growth differences of CD26A54_R compared to CD26A54_S and VLOO13

**Figure 1:**
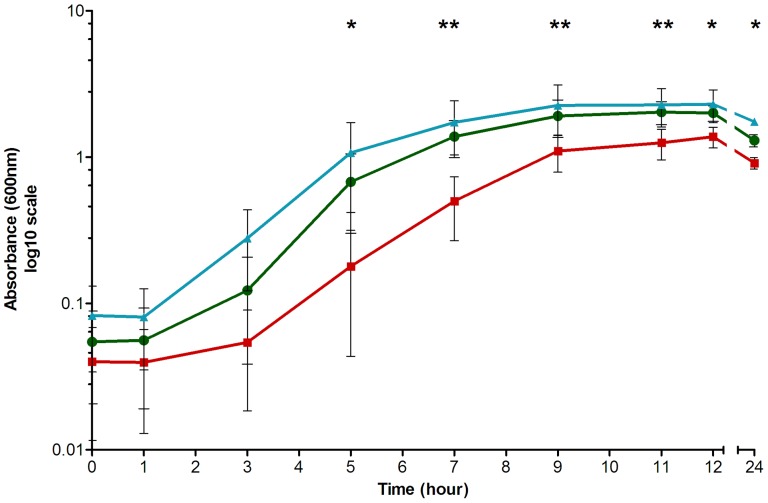
Growth of *C. difficile* in BHI broth. CD26A54_R, the metronidazole resistant strain (□) demonstrated aberrant growth compared to VLOO13 (□) at all time points between 5 and 24 hours (*p*<0.05*). However, the CD26A54_S strain (□) only had a significantly greater cell density than CD26A54_R during late-log and early stationary time points (7–11 hours, *p*<0.05**). There was no significant difference observed between VLOO13 and CD26A54_S throughout the growth curve.

CD26A54_R was observed to have a significantly reduced cell density in BHI broth compared to VLOO13 at all time points from 5–24 hours growth (*p*<0.05) as well as CD26A54_S from 7–11 hours (*p*<0.05). In contrast, there was no significant difference between the growth of CD26A54_S and VLOO13 throughout the entire time course (Figure 1).

When grown on standard BAKHS agar plates, all three *C. difficile* strains used in the study demonstrated similar colony size variability at 48 hours. Attempts were made to separate the colonies by size to differentiate characteristics potentially associated with colony dimensions, however size variability was still observed regardless of the original single colony selected (unpublished data).

The quantitation of spores relative to vegetative cells within BHI broth was performed at 12, 24 and 48 hours post inoculation. Experimental optimization of the spore enumeration protocol was attempted using different shocking methods to select for spores and various media formulations for colony recovery. The cell quantities were inconsistent using these different methods, however a consistent trend was observed. Specifically, the relative percentage of spores increased for CD26A54_S and VLOO13, but remained at or near zero for the metronidazole resistant strain, CD26A54_R. The relative percentage of spores increased over time for both CD26A54_S (1.4%, 12.0%, 42.2%) and VLOO13 (3.2%, 21.7%, 87.2%) at 12, 24 and 48 hours, respectively. Conversely, the percentage of spores in the CD26A54_R culture remained at or near zero (0%, 0.0025%, 0%) for all three time points, respectively.

### Bacterial ultrastructure was altered in both CD26A54 strains

**Figure 2:**
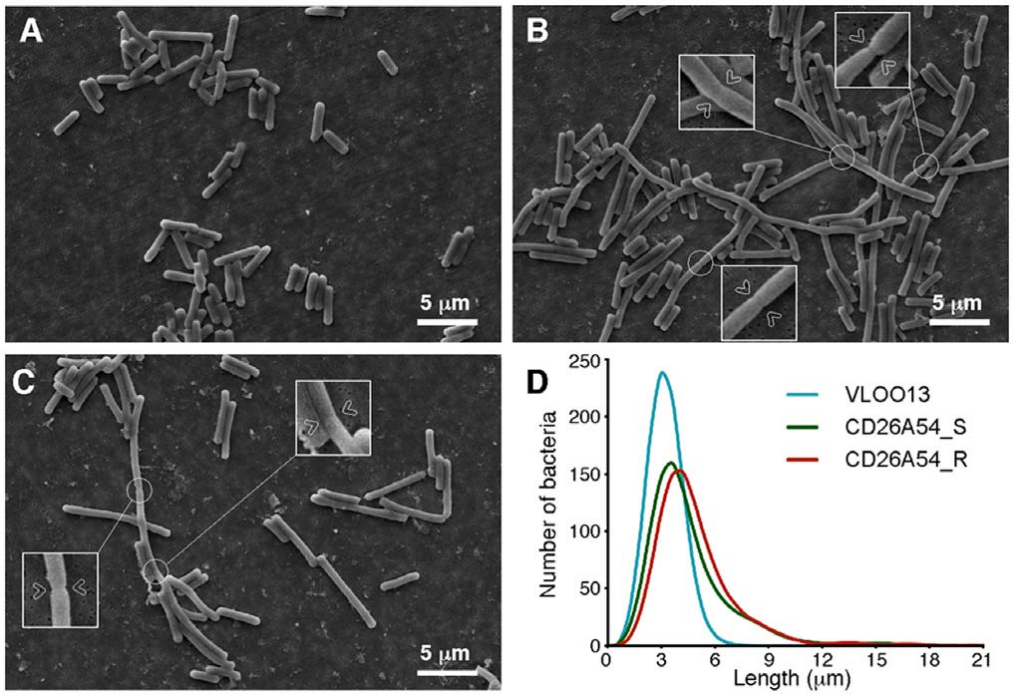
Scanning electron microscopy of *C. difficile*. SEM images of VLOO13 (A), CD26A54_S (B) and CD26A54_R (C). (D) Histograms of calculated bacterial length are presented; they demonstrate the cell length variation that exists across the three *C. difficile* specimens that were imaged by SEM. The insets with arrows in panels (B, C) highlight the septum which partially forms between adjacent cells. Cell separation appears to be impaired resulting in the longer phenotype.

Bacterial ultrastructure was examined by scanning electron microscopy (SEM). The results of this analysis are presented in Figure 2. The initial survey of these images revealed that wild type strain VLOO13 (panel A) bacilli were visibly shorter than the CD26A54_S (panel B) and CD26A54_R (panel C) strains of *C. difficile*. However, statistical comparison of computed bacterial lengths by Kruskal-Wallis ranked sum test revealed all three strains significantly differed from each other (*p*<0.0001). Length of the bacilli were measured, with the results presented as (median ± interquartile difference); CD26A54_R (4.0±2.1 µm, N = 1004), CD26A54_S (3.6±2.1 µm, N = 1002) and VLOO13 (2.8±0.9 µm, N = 1021). All strains tested had the same approximate diameter, suggesting no differences in cell wall thickness. These results are plotted as a histogram in Figure 2 (panel D). The data suggest that both CD26A54_R and CD26A54_S populations contain shorter single cells comparable to VLOO13 in addition to longer bacilli, which appeared to be adjoining cells that have not separated properly. The septum can be visualized at intervals within these long structures (arrows in panel B and C).

### The CD26A54 strains are resistant to multiple antibiotics

Following the isolation of *C. difficile* from the stool sample, the MIC value of strain CD26A54 to metronidazole was 256 mcg/ml by agar dilution and 32 mcg/ml by Etest®. Retesting of the subpopulation that was immediately frozen, CD26A54_S demonstrated an MIC of 2 mcg/ml towards metronidazole (by Etest®) while the strain that had been perpetually maintained on BAKHS+8 mcg/ml metronidazole had an MIC value of 32 mcg/ml.

Antibiotic susceptibility for a panel of 6 antibiotics was performed on the CD26A54_R, CD26A54_S and VLOO13 strains by Etest® strip (Table 2). The full panel of antibiotic susceptibilities was performed in duplicate, while metronidazole susceptibility was tested in 11 independent experiments. Quality control MIC values (ATCC control strains) were consistently within acceptable range for all susceptibility experiments. Based on the interpretive criteria for resistance provided by the Etest® manufacturer's guidelines, all three strains, CD26A54_S, CD26A54_R and VLOO13 were resistant to moxifloxacin (MIC>32 mcg/ml) while only CD26A54_R and CD26A54_S were above the resistant breakpoint for clindamycin (MIC = 8 and MIC = 6 mcg/ml, respectively). All three strains tested were susceptible to vancomycin, rifampicin and tigecycline.

**Table 2:** Antibiotic susceptibility results of *C. difficile* strains by Etest®.

Strain	Metronidazole	Clindamycin	Vancomycin	Rifampicin	Moxifloxacin	Tigecycline
CD26A54_R	12–256	8	0.75	0.003	>32	0.094
CD26A54_S	2–12	6	0.75	0.004	>32	0.125
VLOO13	0.38–1.5	2	0.75	0.004	>32	0.125
*B. fragilis* ATCC25285	0.19–0.25	1.5	*N/A*	*N/A*	0.38	*N/A*
*E. faecalis* ATCC29212	*N/A*	*N/A*	4	1.5	*N/A*	0.75
*S. aureus* ATCC29213	*N/A*	*N/A*	2	*N/A*	*N/A*	*N/A*
*E. coli* ATCC25922	*N/A*	*N/A*	*N/A*	*N/A*	0.064	*N/A*

N/A = not applicable as a quality control strain for this specific antibiotic.

**Figure 3:**
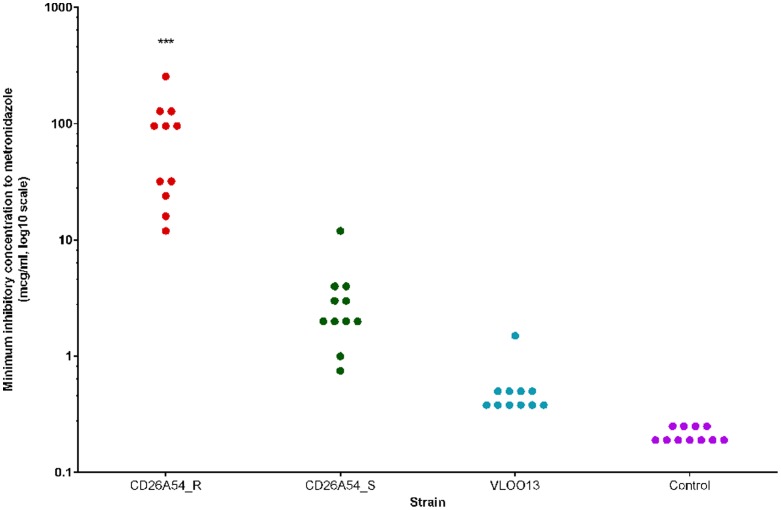
Summary of metronidazole susceptibility results by Etest®. Susceptibility to metronidazole was repeatedly tested for a total of *n* = 11 independent experiments to monitor the stability of the resistance phenotype.

Figure 3 illustrates the results of *n* = 11 independent metronidazole Etest® assays that were conducted prior to all experiments performed to monitor variation and stability of metronidazole susceptibility. Using the Kruskal-Wallis ranked sum test, the median MIC of CD26A54_R was significantly greater compared to CD26A54_S and VLOO13 (*p*<0.001). The range of MIC values for CD26A54_R was 12–256 mcg/ml, spanning 4 doubling dilutions, with the median and mode (most frequent value) being 96 mcg/ml. The CD26A54_S MIC range was 0.75–12 mcg/ml (3 doubling dilutions), with both the median and mode being 2 mcg/ml. A single MIC value of 12 mcg/ml for CD26A54_S was observed and considered to be an outlier. This value was not excluded as the control strain was within acceptable range and the two remaining test strains demonstrated typical results (compared to other replicates) however this elevated MIC was not reproducible. The range of the wild type, VLOO13 strain was much smaller, only spanning 2 doubling dilutions, 0.38–1.5 mcg/ml; the median and mode were both 0.38 mcg/ml. The control strain, *B. fragilis* consistently tested within CLSI acceptable limits with a range of 0.19–0.25 mcg/ml. It is unclear at this time why there were varying ranges of MIC values as all efforts were made to ensure consistent strain handling protocols and environmental conditions.

### The heteroresistant phenotype towards metronidazole was observed for both CD26A54 subpopulations

We examined heteroresistance towards metronidazole using Etest® strips. At 48 hours, the CD26A54_R strain was classified above the resistant breakpoint (MIC = 32 mcg/ml). Under prolonged incubation for 96 hours, we observed further growth leading to reduction of the ellipse (MIC = 96 mcg/ml). The CD26A54_S isolate was susceptible to metronidazole at 48 hours (MIC = 3 mcg/ml), however slower growing colonies were observed within the ellipse increasing the MIC to 16 mcg/ml at 96 hours. The wild type VLOO13 strain, demonstrated MIC values within the same doubling dilution at both 48 and 96 hours, MIC = 0.38 mcg/ml and 0.5 mcg/ml respectively. The *B. fragilis* control strain MIC values were unchanged between 48 and 96 hours, the MIC values were 0.19 mcg/ml at both time points.

### Genetic macrodiversity observed between sequenced NAP1 strains

**Figure 4:**
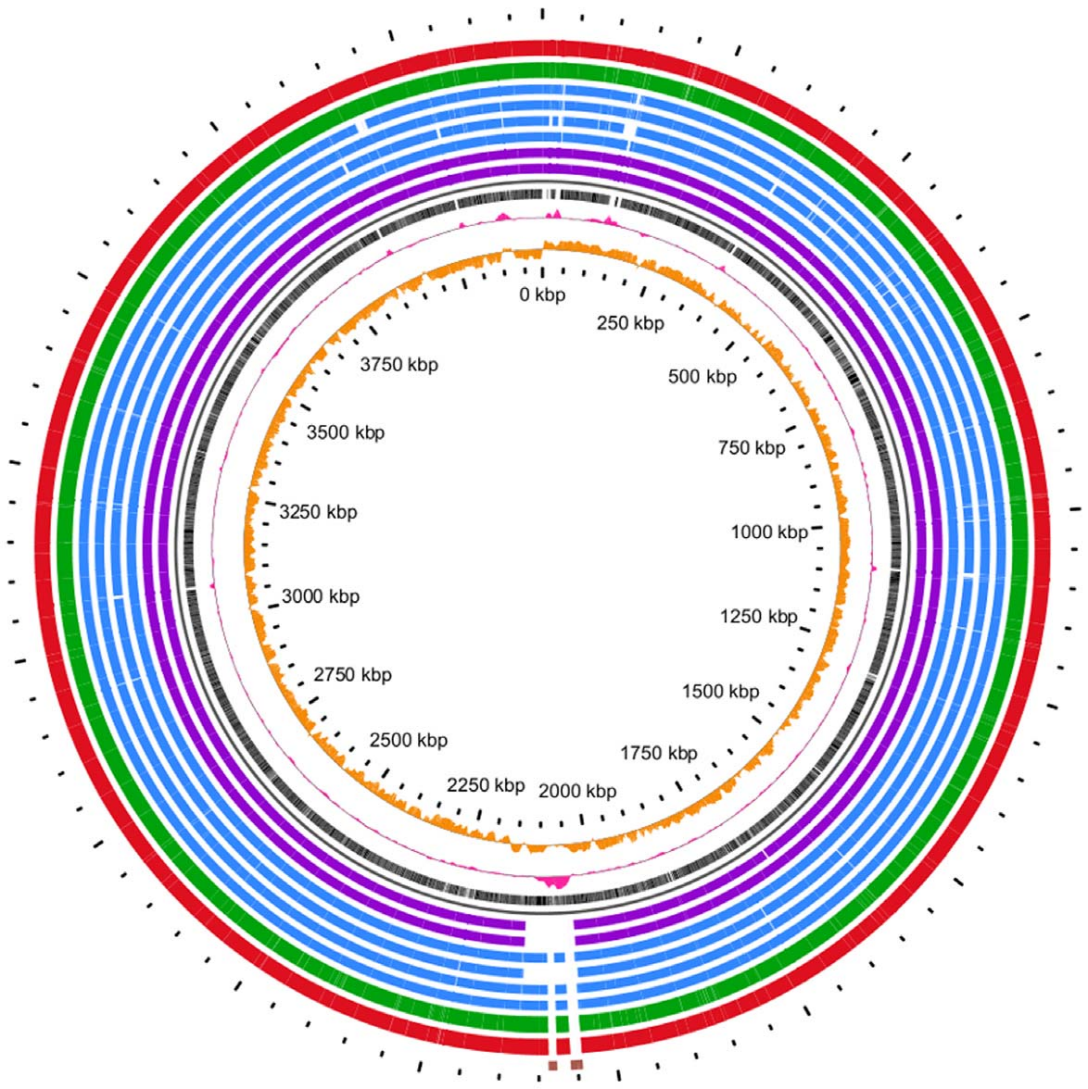
BLAST atlas of NAP1 genomes. BLASTn was used to compare NAP1 genomes listed in Table 1. The rings, listed from inner to outer tracks are as follows: Orange peaks represent GC skew while the pink peaks represent GC content. The innermost black track is the R20291 reference genome. Purple tracks are the historical, pre-epidemic strains, CD196 and BI-1. The blue rings consist of Canadian NAP1 genomes, QCD32g58, QCD-97b34, QCD-37×79 and QCD-66c26. The green is the susceptible CD26A54_S while the red is the resistant subpopulation, CD26A54_R. The outermost ring contains the sequences for the two transposable elements, Tn*6104* and Tn*6106* to confirm their absence in all genomes except R20291.

The macrodiversity of the CD26A54_R and CD26A54_S strains were compared to publically available NAP1 genomes using BLASTn to generate a BLAST atlas (Figure 4). Of particular interest is a region with elevated GC content located just above 2000 kbp which represents the location of four previously identified putative conjugative transposons [56]. The two inner purple rings represent BI-1 and CD196, representing NAP1 strains pre-dating the epidemic NAP1 spread which began in 2003. These two strains are missing all four transposons within this region whereas the other 6 strains, (representing Canadian, post-epidemic NAP1 isolates) are only missing two transposons, Tn*6104* (15.6 kbp) and Tn*6106* (11.3 kbp) (displayed as 2 blocks in the outermost ring in brown) relative to the mapping reference genome R20291. The absence of these two transposons from the publically available NAP1 genomes from Quebec (4 blue concentric rings) has been shown previously [56]. Importantly, this analysis revealed that there was no gain or loss of mobile genetic elements unique to the CD26A54_R strain and that loss of Tn*6104* and Tn*6106* is common amongst Canadian NAP1 strains from both British Columbia and Quebec. As metronidazole resistance was not reported for any of the publically available genomes included in this study [48], [57], [58], we infer that the metronidazole resistant phenotype observed with the CD26A54_R strain did not result from recently acquired or excised genomic content.

### Genetic variation between CD26A54_S and CD26A54_R is at the SNP level

There were 64 total variants identified for the CD26A54_R strain compared to only 45 total variants of the CD26A54_S strain, each in relation to the publically available reference R20291 (NC_013316.1) genome. There were 20 variants, unique to CD26A54_R within CDS (coding DNA sequence) including 17 SNP and 3 indel while CD26A54_S only contained 3 variants, 2 SNP and 1 indel (Table 3). Additionally, there were 23 variants that were common to both CD26A54_R and CD26A54_S compared to the reference sequence, listed in Table 4. In both Tables 3 and 4, the variant confidence is shown in the last three columns with PCR sequence confirmation and/or Illumina coverage and frequency of the variant base call. PCR was performed on 8 genes of interest and included genomic DNA from VLOO13 for comparison. For all 8 genes, the VLOO13 nucleotide sequences were found to be 100% identical to as the reference R20291 sequence. It was observed that all indel variants caused coding frameshift mutations, which are more likely detected (than point mutations) to affect the protein function. Of particular interest were CDS variants in CD26A54_R, located within a putative transcription antiterminator, an oxygen-independent coproporphyrinogen III oxidase gene (*hemN*), a putative N-acetylmuramoyl-L-alanine amidase gene and detected in CD26A54_S in a GntR family transcriptional regulator. Additionally, there were frameshift mutations common to both strains in the anti-sigma factor (*rsbW*), an ATP-dependent helicase gene, a putative nitroreductase gene and a transcriptional regulator. The distinction between variants unique to each strain versus those common to both is noteworthy because although only CD26A54_R is considered resistant by standard CLSI guidelines, CD26A54_S does have reduced susceptibility towards metronidazole and both share phenotype differences which distinguish them from the VLOO13 wild type strain. There were also 21 and 19 intergenic variants discovered within the CD26A54_R and CD26A54_S strains, respectively, however these occurred ≥100 bp from any CDS; they were not listed in the Table 3 or 4.

**Table 3:** Summary of genetic variation within coding regions relative to the reference R20291 genome unique to either CD26A54_R or CD26A54_S.

R20291 Reference		CD26A54_R		CD26A54_S		Gene	Predicted product	PCR^1^	Coverage^2^	Frequency of
Position	Sequence	Variant	AA	Variant	AA	name				variant (%)^3^
920174	A	ATG	Asn219fs	–	–		putative transcription antiterminator		150	96
969792	T	C	SYN	–	–		putative membrane protein	Y	295	100
1220387	G	A	Val113Ile	–	–	*hom1*	homoserine dehydrogenase		373	93.3
1268654	C	A	Thr99Asn	–	–	*spoOA*	stage 0 sporulation protein A	Y	170	87.1
1337528	G	A	Ala30Thr	–	–	*topA*	DNA topoisomerase 1		225	87.1
1353228	G	A	Glu41Lys	–	–	*fur*	ferric uptake regulation protein	Y	339	100
1481407	T	C	SYN	–	–		putative oligopeptide transporter		195	88.2
1487300	G	A	Leu283Phe	–	–		GntR family transcription regulator		85	100
1503742	C	A	Asp254Tyr	–	–		putative signalling protein		340	100
1783253	G	A	Asp593Asn	–	–		sodium ABC extrusion transporter permease		181	100
1890570	C	T	Ser328Phe	–	–	*thiH*	thiamine biosynthesis protein	Y	325	94.5
2160266	T	G	Ser308Ala	–	–		peptidase		319	100
2516619	T	G	Asp259Ala	–	–	*cspC*	putative germination-specific protease	Y	321	99.4
2759453	T	TA	Tyr214fs	–	–	*hemN*	oxygen-independent coproporphyrinogen III oxidase	Y	179	85.5
2955260	C	T	Ala229Thr	–	–	*glyC*	glycerol-3-phosphate dehydrogenase (NAD(P)+)		243	64.6
2995946	G	T	Ala334Glu	–	–		pseudogene		486	52.7
3014935	C	T	Gly423Glu	–	–	*nifJ*	pyruvate-flavodoxin oxidoreductase	Y	362	100
3120023	GT	G	Lys4fs	–	–		putative N-acetylmuramoyl-L-alanine amidase		295	74.6
3277693	T	C	SYN	–	–		altronate hydrolase		431	95.1
4159729	G	A	Leu646Phe	–	–		diguanylate phosphodiesterase		125	100
2688508	T	–	–	A	Leu701Ile		DNA topoisomerase		338	99.7
3473445	AT	–	–	A	Asp76fs		GntR family transcriptional regulator		183	84.2
4147900	A	–	–	G	SYN		hypothetical protein		270	98.9

1 Wet lab confirmation of variant by sequenced PCR products using target specific primers.

2 Fold coverage of Illumina sequences at this position.

3 Frequency of the variant, presented as a percentage for comparison.

**Table 4:** Variants within coding regions common to both CD26A54_R and CD26A54_S with reference to R20291.

R20291 Reference		CD26A54_R & S		Gene	Predicted product	PCR^1^	Coverage^2^	Frequency
Position	Sequence	Variant	AA	name				of variant (%)^3^
6417	G	T	Ala118Ser	*gyrA*	gyrase A		457	100
9661	C	CA	Ala71fs	*rsbW*	anti-sigma factor protein		269	91.5
120932	C	A	SYN	*rpoA*	DNA directed RNA polymerase subunit A		411	99.8
1116866	G	T	Ala53Ser		putative membrane protein (pseudogene)		207	100
1568676	C	A	Gln138Lys		ruberythrin		486	100
1876233	A	C	Val390Gly		putative arsenical pump membrane protein		288	100
2134201	A	C	Glu70Ala		lipoprotein		227	100
2297662	T	C	Glu110Gly		putative lipid kinase		259	99.2
2297977	A	T	Leu5Stp		conserved hypothetical protein		300	100
2531178	A	G	SYN		PTS system, EIIc component		323	100
2743315	TA	T	Phe255fs		ATP-dependent helicase		179	92.7
2840701	C	A	Ser414Ile		membrane-associated 5′-nucleotidase		286	100
2947240	G	T	Ala217Glu		hypothetical protein		355	100
2955330	A	G	SYN	*glyC*	glycerol-3-phosphate dehydrogenase		546	100
2976764	G	A	Thr30Ile	*murD*	UDP-N-acetylmuramoyl-L-alanine:D-glutamate ligase		250	100
3304975	C	T	Glu72Lys	*ntpC*	V-type ATP synthase subunit C		88	100
3437506	T	G	SYN		PTS system transporter subunit IIC		274	100
3489680	C	A	Met193Ile		PTS system transporter subunit IIBC		508	100
3531583	C	T	Val389Ile		PTS system transporter subunit IIABC		245	100
3814691	TC	T	Glu136fs		putative nitroreductase	Y	184	100
3815543	TG	T	Trp99fs		transcriptional regulator		373	91.7
3832901	T	C	SYN		RNA methyltransferase		370	99.2
3913225	A	C	SYN		transposase		30	100

1 Wet lab confirmation of variant by sequenced PCR products using target specific primers.

2 Fold coverage of Illumina sequences at this position.

3 Frequency of the variant, presented as a percentage for comparison.

**Figure 5:**
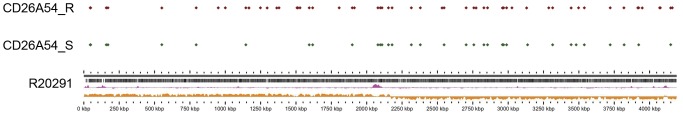
Variant distribution of both CD26A54_R and CD26A54_S relative to the reference sequence R20291. The total SNP and indel variants for the CD26A54_R (red diamonds) and the CD26A54_S (green diamonds) are plotted along the R20291 reference genome. The reference sequence tracks include CDS (black bars), GC content (pink peaks) and GC skew (orange peaks).

Distribution of all detected variants (within CDS and intergenic) relative to R20291 are illustrated in Figure 5. The reference genome R20291 (NC_013316.1) is denoted along the bottom with the CDS (black bars), GC content (pink track) and GC skew (orange track) plotted. Most regions with elevated GC content (pink peaks) correspond to ribosomal coding regions with the exception of a large peak between 2000 kbp and 2250 kbp corresponding to the variably present four putative conjugative transposons [56]. Figure 5 reveals that the genetic variants of both strains are randomly distributed throughout the entire genome. Moreover, the CD26A54_R isolate contained more variants, likely attributed to the extensive laboratory passaging of this isolate in the presence of sub-inhibitory metronidazole.

## Discussion

We have presented four phenotypes associated with the stable, metronidazole-resistant CD26A54_R subpopulation: (i) aberrant growth in liquid media, (ii) attenuated cell wall separation (iii) lack of spore production by 48 hours and (iv) heteroresistance or more accurately, a slower growing subpopulation that increases the metronidazole MIC. By standard diagnostic methods CD26A54_S is considered susceptible to metronidazole, however this strain shares some characteristics with its resistant counterpart, specifically attenuated cell separation and heteroresistance against metronidazole thus also distinguishing it from the wild type VLOO13. We will discuss how whole genome sequence data may explain these phenotypes however further investigation is needed to understand the role of these changes in metronidazole resistance and how this impacts pathogenicity.

In BHI broth, CD26A54_R appeared to have a prolonged lag phase followed by reduced cell density, particularly during the late-log to early stationary phases. Even at 24 hours post-inoculation, CD26A54_R never reached the same cell density as either CD26A54_S or VLOO13. This may suggest CD26A54_R encountered a nutrient limitation in the media that was not exogenously required by the other strains, enabling the other two to reach a higher cell density. Genetically, there are two variants that may have contributed to the aberrant growth observed, namely the frameshift mutation in the *hemN* gene, whose product is involved haem biosynthesis and *thiH*, encoding a protein involved in thiamine biosynthesis. There are additional variants found in CD26A54_R that are associated with electron transport, such as glycerol-3-phosphate dehydrogenase (*glyC*) and the pyruvate-flavodoxin oxidoreductase (*nifJ*). Disruption of electron transport alters both the energy production and intracellular redox potential which influence the efficiency of metronidazole entry and activation [59].

Deficiencies leading to disruptions in electron transport have been previously attributed to small colony variants (SCV) in other bacterial genera. Some general characteristics of SCV include smaller colonies on agar, slower growth, decreased respiration, attenuated cell separation and antibiotic resistance; additionally, they are often associated with persistent or recurrent infections [60].We have observed by SEM that both CD26A54_R and CD26A54_S demonstrate elongated cells and incomplete cell separation which is similar to previous reports of *Enterococcus faecium* SCV [61], [62] and *Staphylococcus aureus* SCV [63]. The shared characteristics of previously described SCV and the CD26A54 subpopulations such as aberrant growth, attenuated cell separation and antibiotic resistance have been demonstrated, however we have not observed smaller colonies on non-selective agar.

Resistance to additional antibiotics was tested to assess possible disruptions in metabolic pathways such as protein or cell wall biosynthesis that could contribute to altered cell morphology and aberrant growth however the MIC values of CD26A54_R were similar to one or both of the metronidazole-susceptible strains. A high prevalence of moxifloxacin and clindamycin resistance among ribotype 027 strains has been reported previously (97.5% and 47.5% respectively), although no observations of metronidazole resistance or altered cell morphology were reported [15]. We have a few hypotheses with respect to our observations that CD26A54_R did not produce spores by 48 hours whereas CD26A54_S and VLOO13 both demonstrate an increasing proportion of spores. There are multiple methods in the literature to study sporulation of *C. difficile* isolates (reviewed in [64]). We did attempt this analysis with various media formulations and methods to shock the culture for spore selection, and although total numbers did vary across replicates and across individual experiments, we observed a consistent trend that no spores were ever counted in the CD26A54_R culture up to 48 hours. Within the CD26A54_R genome there is a point mutation in the stage 0 sporulation protein A gene (*spo0A*) which is a major regulator of the sporulation process [65], as well as a nonsynonymous variant in the *cspC* gene which encodes a putative germination-specific protease. Further experimentation is needed to investigate whether this data demonstrates a lack or delay of spore production owing to disruption of the spore initiation pathway (via *spoOA* mutation) or conversely owing to disruption in the germination process resulting from the *cspC* mutation; causing the inability of spores to germinate efficiently. We speculate that disruption of the sporulation process could prolong the vegetative state resulting in prolonged expression of virulence factors during host infection. Importantly however, reduced propensity for spore generation and/or germination should render CD26A54_R less transmissible in hospital or long-term care facility settings as vegetative cells are more susceptible to regular cleaning methods.

In addition to the phenotypic and genotypic changes described above, we identified genetic mutations similar to those of other bacterial genera which have been shown to confer metronidazole resistance. The CD26A54_R strain possesses a point mutation in the *fur* gene of which encodes the ferric uptake regulator, Fur. Disruption of the *fur* gene has been associated with metronidazole resistance in *H. pylori* by altering binding of Fur to superoxide dismutase thereby reducing oxidative stress within the cell [66]. Both CD26A54_R and CD26A54_S contain a frameshift mutation in the *rsbW* gene which encodes an anti-sigma factor associated with σ^B^, which plays a central role in stress responses [67]. We speculate that alteration of *rsbW* may similarly enhance the ability of the bacterium to reduce oxidative stress caused by metronidazole. Both strains also possess a frameshift mutation in a putative nitroreductase gene which may alter the requisite activation of metronidazole in the cell [59]. The association between nitroreductases and metronidazole resistance has been described previously for both *B. fragilis* and *H. pylori* [29], [31], [33]–[35].

As discussed above, there are numerous possible mechanisms to explain the metronidazole resistance observed in the current study. In the literature there are multiple mechanisms for metronidazole resistance described within a species suggesting resistance can occur via different pathways and can be a multifactorial process. In the present study we presented phenotypic observations and genomic analyses of a stable, metronidazole resistant isolate of *C. difficile* in the aim of providing a foundation for future experimental investigation to elucidate the mechanisms of resistance and ultimately translate the knowledge to improve clinical diagnosis and treatment of CDI.
